# Supplementary material for: Competition for Nitrogen Resources: An Explanation of the Effects of a Bioprotective Strain Metschnikowia pulcherrima on the Growth of Hanseniaspora Genus in Oenology
Source: Foods. 2024 Feb 27;13(5):724. doi: 10.3390/foods13050724 (PMC10930766; doi:10.3390/foods13050724)
Supplement: Supplementary file 1 [file foods-13-00724-s001.zip › foods-2857400-supplementary.pdf]

Table S1: Synthetic must (MS300) composition

Compounds	g/L	Compounds	g/L
Glucose	100	NH_4Cl	0,46
Fructose	100	Tyrosine	0,018326
(DL)-malic acid	6	Tryptophane	0,179333
citric acid	6	Isoleucine	0,032725
KH_2PO_4	0,75	Aspartic acid	0,044506
K_2SO_4	0,5	Glutamic acid	0,120428
$MgSO_4,7H_2O$	0,25	Arginine	0,374374
$CaCl_2$, $2H_2O$	0,155	Leucine	0,048433
NaCl	0,2	Threonine	0,075922
$MnSO_4$, H_2O	0,004	Glycine	0,018326
$ZnSO_4$, $7H_2O$	0,004	Glutamine	0,505274
$CuSO_4$, $5H_2O$	0,001	Alanine	0,145299
KI	0,001	Valine	0,044506
$CoCl_2$, $6H_2O$	0,0004	Methionine	0,031416
H_3BO_3	0,001	Phenylalanine	0,037961
$(NH_4)_6Mo_7O_{24}$, $4H_2O$	0,001	Serine	0,07854
Myo-inositol	0,02	Histidine	0,032725
Calcium pantothenate	0,0015	Lysine	0,017017
Thiamine, hydrochloride	0,00025	Cysteine	0,01309
Nicotinic acid	0,002		
Pyridoxine	0,00025		
Biotin	0,000003		
Ergosterol	0,003		
Oleic acid	0,001		
Tween 80	0,1		

Table S2: Eluant gradient for amino acids analyses by HPLC.

Time (min)	Mobile phase A (%)	Mobile phase B (%)
0.00	98	2
0.25	98	2
9.40	47	53
9.50	0	100
16.50	0	100
16.51	98	2
18.50	98	2

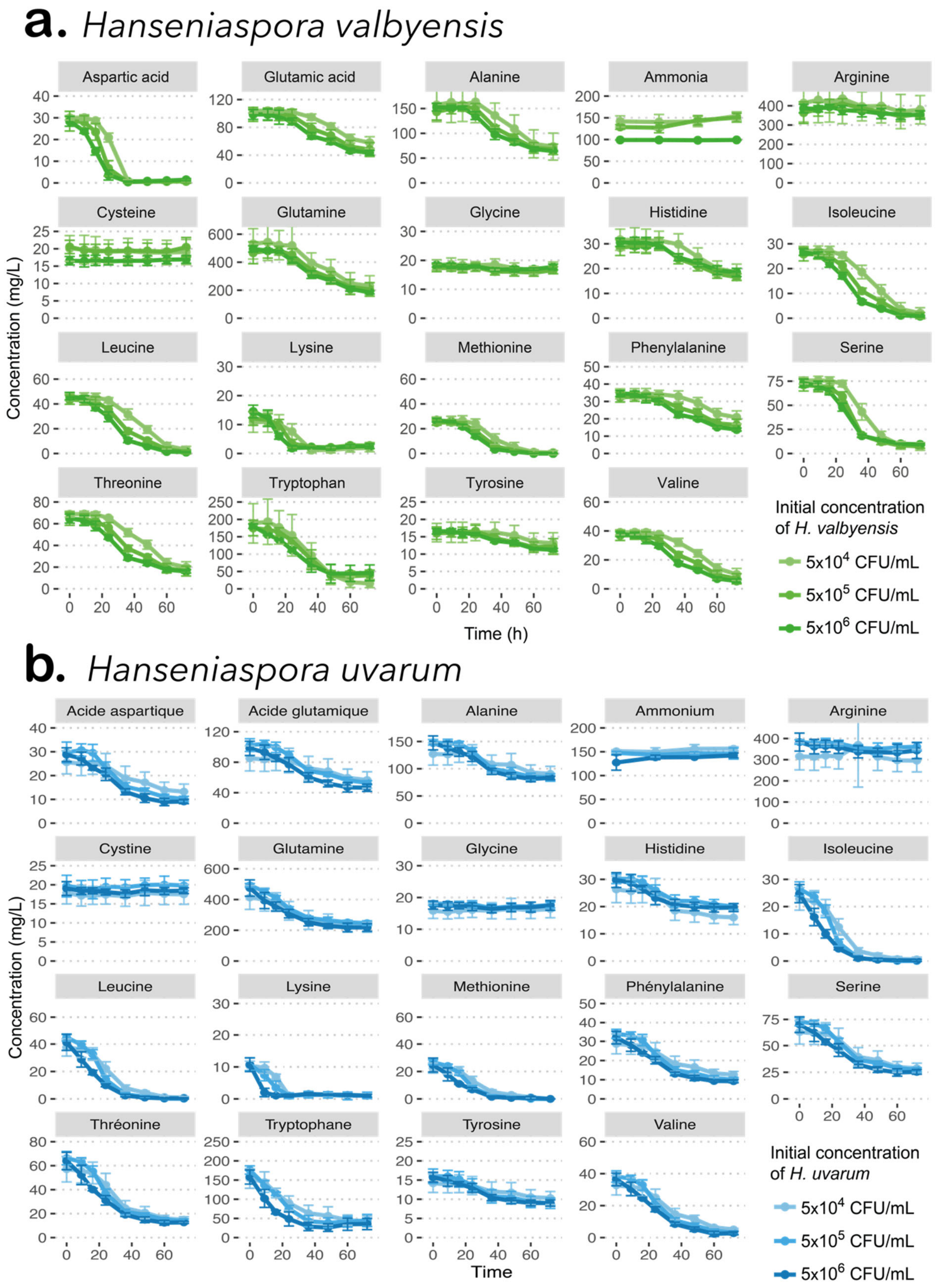
a. Hanseniaspora valbyensis
Aspartic acid
Glutamic acid
Alanine
Ammonia
Arginine
Cysteine
Glutamine
Glycine
Histidine
Isoleucine
Leucine
Lysine
Methionine
Phenylalanine
Serine
Threonine
Tryptophan
Tyrosine
Valine
Concentration (mg/L)
Time (h)
Initial concentration of H. valbyensis
5x10^4 CFU/mL
5x10^5 CFU/mL
5x10^6 CFU/mL
b. Hanseniaspora uvarum
Acide aspartique
Acide glutamique
Alanine
Ammonium
Arginine
Cystine
Glutamine
Glycine
Histidine
Isoleucine
Leucine
Lysine
Methionine
Phénylalanine
Serine
Thréonine
Tryptophane
Tyrosine
Valine
Concentration (mg/L)
Time
Initial concentration of H. uvarum
5x10^4 CFU/mL
5x10^5 CFU/mL
5x10^6 CFU/mL

c. *M. pulcherrima*

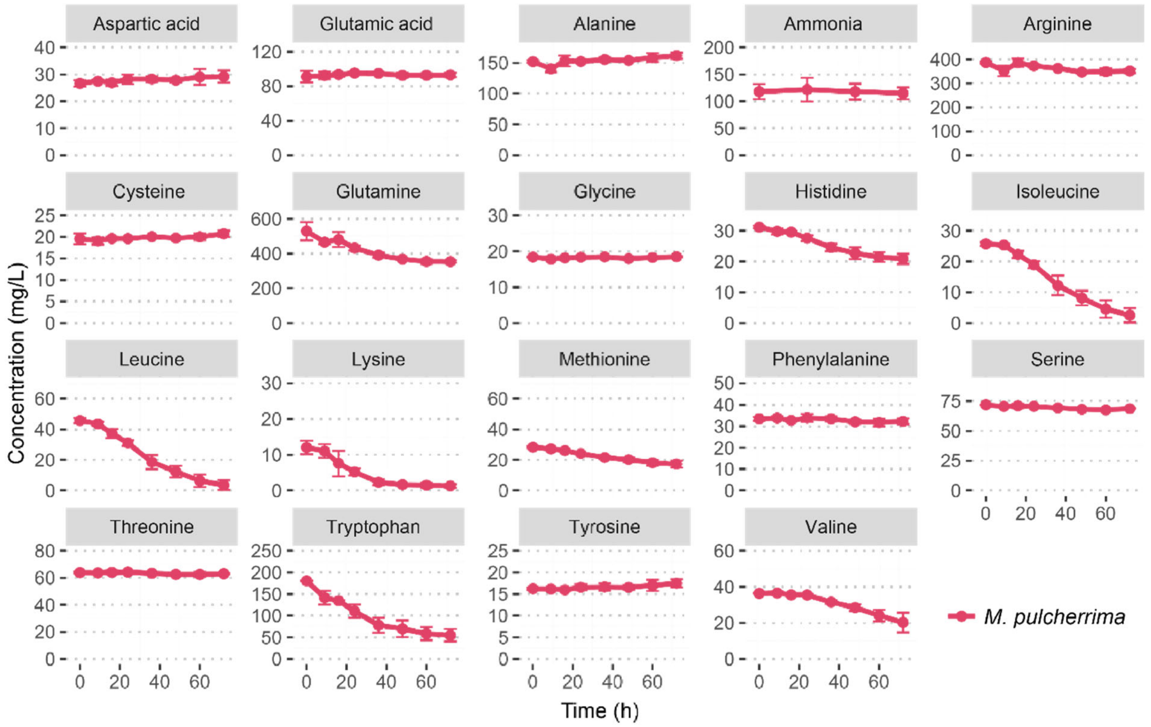

Figure S1: Nitrogen consumption of (a) *H. valbyensis,* (b) *H. uvarum* and (c) *M. pulcherrima* MCR24 in single culture for 72 hours of growth at 20 °C.

Table S3: Percentage of consumption of each amino acid and ammonium for each condition at different times of growth. Color intensity represents consumption intensity. The white color indicates that no values are available.

	Hanseniaspora uvarum												*Hanseniaspora valbyensis*												*M. pulcherrima*			
	5.10^4 CFU/mL				5.10^5 CFU/mL				5.10^6 CFU/mL				5.10^4 CFU/mL				5.10^5 CFU/mL				5.10^6 CFU/mL				5.10^5 CFU/mL			
	9h	24h	48h	72h	9h	24h	48h	72h	9h	24h	48h	72h	9h	24h	48h	72h	9h	24h	48h	72h	9h	24h	48h	72h	9h	24h	48h	72h
Aspartic acid	3	6	33	48	1	21	54	65	6	29	63	68	1	30	100	98	3	77	98	98	16	87	97	95	5	1	0	0
Glutamic acid	1	3	20	33	1	12	37	48	5	22	47	53	1	2	22	44	1	3	31	51	1	10	39	56	2	0	1	2
Alanine	2	1	16	28	2	11	37	44	6	17	41	43	0	0	31	55	1	3	37	52	1	11	46	58	5	1	1	3
Ammonium		3	1	0		3	1	3		2	1	0		6	3	2		3	0	0		0	1	0		3	1	3
Arginine	2	1	1	6	2	2	9	6	6	6	12	10	0	0	3	8	0	1	4	6	1	2	7	9	6	4	10	14
Cystine	4	3	1	2	4	3	2	3	4	5	3	4	3	5	6	5	6	7	6	3	2	2	1	0	3	3	1	1
Glutamic acid	1	3	20	33	1	12	37	48	5	22	47	53	1	2	22	44	1	3	31	51	1	10	39	56	2	0	1	2
Glutamine	4	21	38	47	8	31	46	50	17	35	52	54	1	4	36	57	0	7	38	56	2	16	46	63	8	17	29	35
Glycine	2	0	1	0	1	2	5	3	2	3	5	2	0	1	6	6	1	1	8	5	2	3	8	4	2	1	1	0
Histidine	2	3	32	39	1	14	27	34	4	22	33	34	1	1	22	42	3	1	25	41	1	4	27	39	4	12	25	32
Isoleucine	4	45	91	97	11	74	99	100	35	81	98	99	1	6	60	94	2	18	74	96	4	36	85	97	4	24	63	84
Leucine	4	41	88	97	12	68	97	100	31	76	97	99	1	6	58	94	2	18	76	97	5	36	87	98	5	28	68	87
Lysine	6	90	87	89	32	93	89	92	83	90	87	91	2	30	101	93	4	70	81	76	17	87	87	83	9	50	81	86
Methionine	4	41	87	100	10	68	96	100	28	72	97	100	1	8	82	97	2	27	89	100	6	47	94	100	3	13	25	35
Phenylalanine	2	14	42	56	3	31	61	70	12	37	66	71	1	1	15	40	1	3	29	49	1	13	42	60	1	2	4	5
Serine	2	11	42	54	3	26	54	62	11	35	60	64	1	4	76	90	2	17	83	89	4	34	81	87	2	3	4	5
Threonine	2	23	64	74	5	44	71	79	19	51	76	80	1	5	41	71	2	13	55	75	3	27	63	75	2	2	2	2
Tryptophane	6	42	64	73	22	57	78	75	36	70	83	78	1	19	80	97	5	20	77	78	13	38	76	75	16	39	61	70
Tyrosine	2	4	17	27	1	14	35	44	6	18	39	43	1	1	5	19	3	2	16	24	0	5	20	32	2	1	1	0
Valine	3	23	65	84	4	43	80	92	17	51	85	92	1	3	37	75	2	7	52	81	2	21	65	86	3	4	19	37

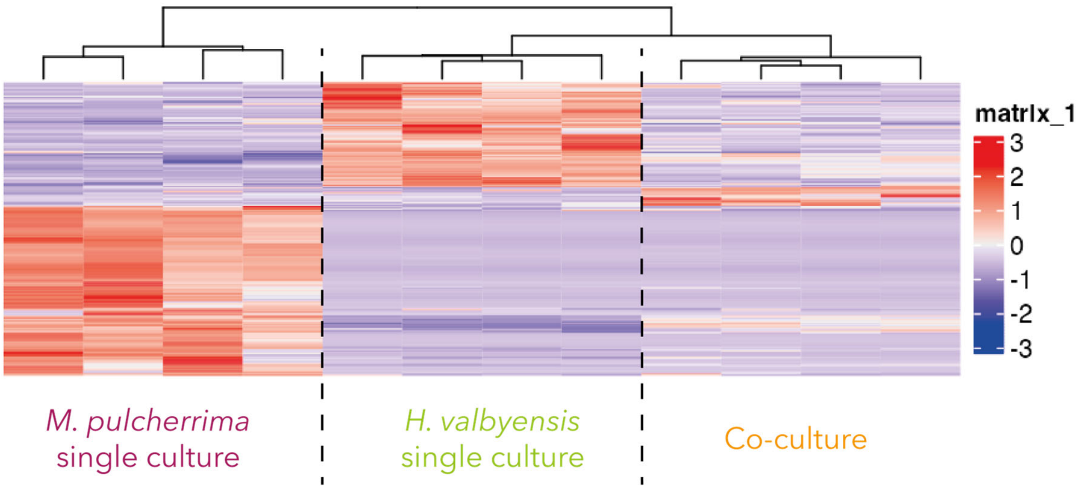

Figure S2: Heat map and hierarchical clustering of each condition according to their biomarkers (biomarkers detailed in Figure 4).
